# Impact of congenital uterine anomalies on reproductive outcomes of IVF/ICSI-embryo transfer: a retrospective study

**DOI:** 10.1186/s40001-023-01544-2

**Published:** 2024-01-11

**Authors:** Jia Kang, Jie Qiao

**Affiliations:** 1 Center for Reproductive Medicine, Department of Obstetrics and Gynecology, Peking University Third Hospital, Beijing, 100191 China; 2 National Clinical Research Center for Obstetrics and Gynecology, Peking University Third Hospital, Beijing, China; 3 Key Laboratory of Assisted Reproduction (Peking University), Ministry of Education, Beijing, China; 4 Beijing Key Laboratory of Reproductive Endocrinology and Assisted Reproductive Technology, Beijing, China; 5 Beijing Advanced Innovation Center for Genomics, Beijing, China; 6 Peking-Tsinghua Center for Life Sciences, Peking University, Beijing, China

**Keywords:** Congenital uterine anomalies, Assisted reproductive outcomes, IVF, ICSI, Embryo transfer

## Abstract

**Objective:**

To study the impact of congenital uterine anomalies on reproductive outcomes after in vitro fertilization (IVF)/intracytoplasmic sperm injection (ICSI)-embryo transfer (ET).

**Methods:**

A retrospective study including a total of 865women with congenital uterine anomalies and 865 age and admission time matched controls who underwent the first IVF/ICSI-ET cycle between January 2010 and December 2019 was conducted. Women with uterine anomalies were classified into canalization defect (complete septate uterus and subseptate uterus) and unification defect (unicornuate uterus, bicornuate uterus, and didelphus uterus) according to the processes of abnormal embryological development. Control women were selected by age (± 1.0 year) and admission time (± 6 months) matched with a 1:1 ratio. The reproductive outcomes were compared between women with uterine anomalies and the controls. The primary outcome was live birth; secondary outcomes were clinical pregnancy, ectopic pregnancy, preterm delivery, and spontaneous pregnancy loss.

**Results:**

Compared with women with a normal uterus, women with canalization defects were less likely to experience live birth [84/332 (25.3%) vs 128/332 (38.6%), RR: 0.647, 95% CI 0.513–0.815, P < 0.001]. They also had a lower clinical pregnancy rate [126/332 (38.0%) vs 206/332 (62.0%), RR: 0.829, 95% CI 0.690–0.997, P = 0.046] and experienced a higher first-trimester pregnancy loss rate [25/126 (19.8%) vs 11/206 (5.3%), RR: 2.716, 95% CI 1.393–5.295, P = 0.003]. Compared with women with a normal uterus, women with a unification defect were also less likely to experience live birth [132/533 (24.8%) vs 219/533 (41.1%), RR: 0.713, 95% CI 0.586–0.868, P = 0.001]. Women with a unification defect had lower clinical pregnancy rates [182/533 (34.1%) vs 263/533 (49.1%), RR: 0.813, 95% CI 0.695–0.952, P = 0.010] and increased first-trimester pregnancy loss [36/182 (19.8%) vs 20/263 (7.6%), RR: 3.288, 95% CI 1.776–6.085, P < 0.001]. While uterine anomaly seemed not increase the risk of preterm birth, ectopic pregnancy and second-trimester pregnancy loss.

**Conclusions:**

Both canalization defects and unification defects were associated with lower fertility outcomes, including lower live birth rates, lower clinical pregnancy rates, and higher early miscarriage rates.

**Supplementary Information:**

The online version contains supplementary material available at 10.1186/s40001-023-01544-2.

## Introduction

Congenital uterine anomalies result from embryological abnormal organogenesis, fusion or septal absorption of the Müllerian ducts, which present as hypoplasia/agenesis, unicornuate uterus, bicornuate uterus, didelphus uterus, complete septate uterus, partial septate uterus, arcuate uterus, and diethylstilbestrol-related uterus according to the American Fertility Society (AFS) classification [1, 2]. Uterine anomalies are often diagnosed during an infertility evaluation because most are asymptomatic. The incidence of uterine anomalies in the infertility population is higher than that in the general population. A systematic review estimated the overall prevalence of uterine malformations to be 5.5% in the general population, 8.0% in infertile women, 13.3% in those with a history of miscarriage, and 24.5% in those with miscarriage and infertility [3], suggesting a connection between congenital uterine anomaly with infertility and adverse pregnancy outcomes.

Evidence suggests that uterine anomalies might be associated with adverse obstetrical outcomes, including increased rates of miscarriage, malpresentation, and preterm birth [4, 5], while little is known about the reproductive outcomes after assisted reproduction technology (ART) treatments. Previous studies have focused on the in vitro fertilization (IVF) outcomes of women with congenital uterine anomalies but have reached contradictory results. Previous studies analyzed different malformations all together [6], while the results were skewed by the high proportion of arcuate uterus (e.g., 89.1% and 89.5%), which was once thought to be a minor defect that did not have any effect on pregnancy outcomes. Most studies have focused on only a particular type of uterine anomaly [7–9]. A retrospective study did rigorous work, but they focused solely on the frozen thawed embryo transfer (FET) outcomes of uterine anomaly [10], while the systematic study of fresh embryo transfer (IVF-ET) outcomes has not been studied.

The spectrum of uterine anomalies ranges from those with a minor defect, where the cavity is only partially altered, such as arcuate uterus, to major defects in which the cavity has bilateral horns or is divided into two by septum, including unicornuate, bicornuate, didelphys, partial septate, and total septate uterus [4, 11]. It is also widely accepted that the various types of Müllerian anomalies are associated with varying degrees of adverse pregnancy outcomes, with greater effects being evident in women with more profound defects [12]. Hence, it is necessary to evaluate the assisted reproductive outcomes of patients with congenital uterine anomalies according to specific type. Considering the embryological development of the bilateral Müllerian duct, a didelphys uterus, bicornuate uterus, and unicornuate uterus can be classified as unification defects, which are due to the total or partial absence of fusion of the Müllerian duct. Septate and partial septate uteri can be classified as canalization defects, which are a result of total or partial absence of reabsorption of the septum between bilateral Müllerian ducts [11, 13].

In this retrospective study, we aimed to investigate the impact of canalization defects and unification defects on IVF-ET outcomes.

## Materials and methods

### Ethics declaration

This study was approved and guided by the ethical committee of the Peking University Third Hospital (project: IRB00006761-M2020004). Informed consent was obtained from all participants. All procedures performed in studies involving human participants were in accordance with the ethical standards of the institutional committee and with the 1964 Helsinki declaration and its later amendments or comparable ethical standards.

### Study design, setting, and participants

This retrospective study was conducted at Reproductive center of Peking University Third Hospital, Beijing, China. Patients were recruited retrospectively between January 2010 and December 2019 during their initial assessment for subfertility, defined as failure to achieve pregnancy within 12 months in women younger than 35 years or within 6 months in women older than 35 years [14].

By using the keywords “congenital uterine anomaly”, “septate uterus”, “unicornuate uterus”, “didelphus uterus”, and “bicornuate uterus”, we retrieved the clinical data of patients diagnosed by three-dimensional (3D) transvaginal ultrasound with major congenital uterine anomalies and underwent IVF during January 2010 and December 2019 at Peking University Third Hospital, Beijing, China. Telephone follow-up would be conducted for each couple one month, six months and one year after embryo transplantation to ask about the outcome.

### Variables and measurement

The primary outcome measure was live birth, defined as the birth of a living fetus beyond 22 weeks of gestational age [15]. Secondary outcomes included the followings: (1) clinical pregnancy: a pregnancy diagnosed by ultrasonographic visualization of one or more gestational sacs. It included ectopic pregnancy. (2) Ectopic pregnancy: a pregnancy in which implantation takes place outside the uterine cavity. (3) Preterm birth: a live birth or stillbirth that takes place after at least 28 but before 37 completed weeks of gestational age [16]. (4) Spontaneous pregnancy loss/miscarriage: the spontaneous loss of a clinical pregnancy before 27 completed weeks of gestational age or, if gestational age is unknown, the loss of an embryo/fetus of less than 1000*g* [17]. It is divided into early pregnancy loss and late pregnancy loss. Early pregnancy loss refers to pregnancy loss occurring before 12 weeks; late pregnancy loss is pregnancy loss occurring after 12 weeks. The controlled ovarian hyperstimulation protocols are described in Additional file 1. 

### Diagnosis of uterine anomalies

Two-dimensional (2D) transvaginal ultrasound was routinely performed during the patients’ initial assessment. If a uterine anomaly was suspected, three-dimensional (3D) transvaginal ultrasound was performed to confirm the diagnosis. Uterine malformations were diagnosed based on the classification system originally proposed by the American Fertility Society and subsequently modified according to 3D ultrasound landmarks [1, 18]. The arcuate uterus was sonographically diagnosed when there was a concave fundal indentation with an angle of indentation > 90 degrees. A septate uterus was diagnosed when a septum was demonstrated on the coronal plane that did not extend to the cervix (subseptate uterus) or completely divided the cavity from the fundus to the cervix (total septate uterus), with the central point of the septum at an acute angle (< 90 degrees) and uniform external convexity or with indentation < 10 mm. A bicornuate uterus was defined as two well-formed uterine cornua with an external fundal indentation > 10 mm. Unicornuate with or without rudimentary horn presents as a single well-formed uterine cavity with a single interstitial portion of fallopian tube and concave fundal contour [19–21]. A didelphic uterus was diagnosed as two well-formed cavities with a single interstitial portion of the fallopian tube and two cervices [1, 6].

### Bias and study size

We enrolled subjects with a 3-D ultrasound diagnosis of “uterine anomaly” rather than a clinical diagnosis of “uterine malformation” to minimize selection bias. In addition, we reduced confounding bias by matching control groups with age and admission time, and by adjusting for all possible confounding factors by log binomial regression.

A total of 2173 women diagnosed with major congenital uterine anomalies and underwent IVF during January 2010 and December 2019 were retrieved. Among the 2173 women with congenital uterine anomalies, 1398 women underwent first-cycle controlled ovarian stimulation (COS), and 966 women underwent IVF-ET. One hundred and one women were excluded because of undefined uterine anomalies and endometrial polyps, endometritis, endometrial tuberculosis, and a history of pelvic tuberculosis. Finally, 865 patients with well-defined uterine anomalies who underwent first cycle COS and IVF-ET and without endometrial disease were enrolled. We performed a post-hoc power analysis with the obtained sample size and the results of the primary outcome (live-birth rate), which resulted in a power of 95.5% in canalization group and a power of 99.9% in unification group at an alpha level of 0.05.

### Statistical analysis

All statistical analyses were performed using SPSS 23.0 software (SPSS, Inc., Chicago, IL, USA). Continuous variables are presented as the mean (± SD) or median (quartile 1–quartile 3). Categorical variables are presented as frequencies (percentages). Student’s t test or nonparametric test was used for comparison of continuous variables between the groups. The chi-squared test or Fisher’s exact test, where appropriate, was used for comparisons of categorical variables. We did selection of controls matched by age (± 1.0 year) and admission time (± 6 months) with a 1:1 ratio, using the SPSS-case–control matching function. Multivariate log binomial regression was used to evaluate primary and secondary outcomes. Given that a single-center retrospective study of 17,978 women led by our hospital found that the women's age, body mass index, duration of infertility years, controlled ovarian hyperstimulation protocol, the number of acquired oocytes, and number of transferrable embryos were the prognostic factors that significantly affected the cumulative live birth rate, all models were adjusted for the above factors [22]. Statistical significance was set at a P value < 0.05 (two-sided).

## Results

### Participants

**Fig. 1:**
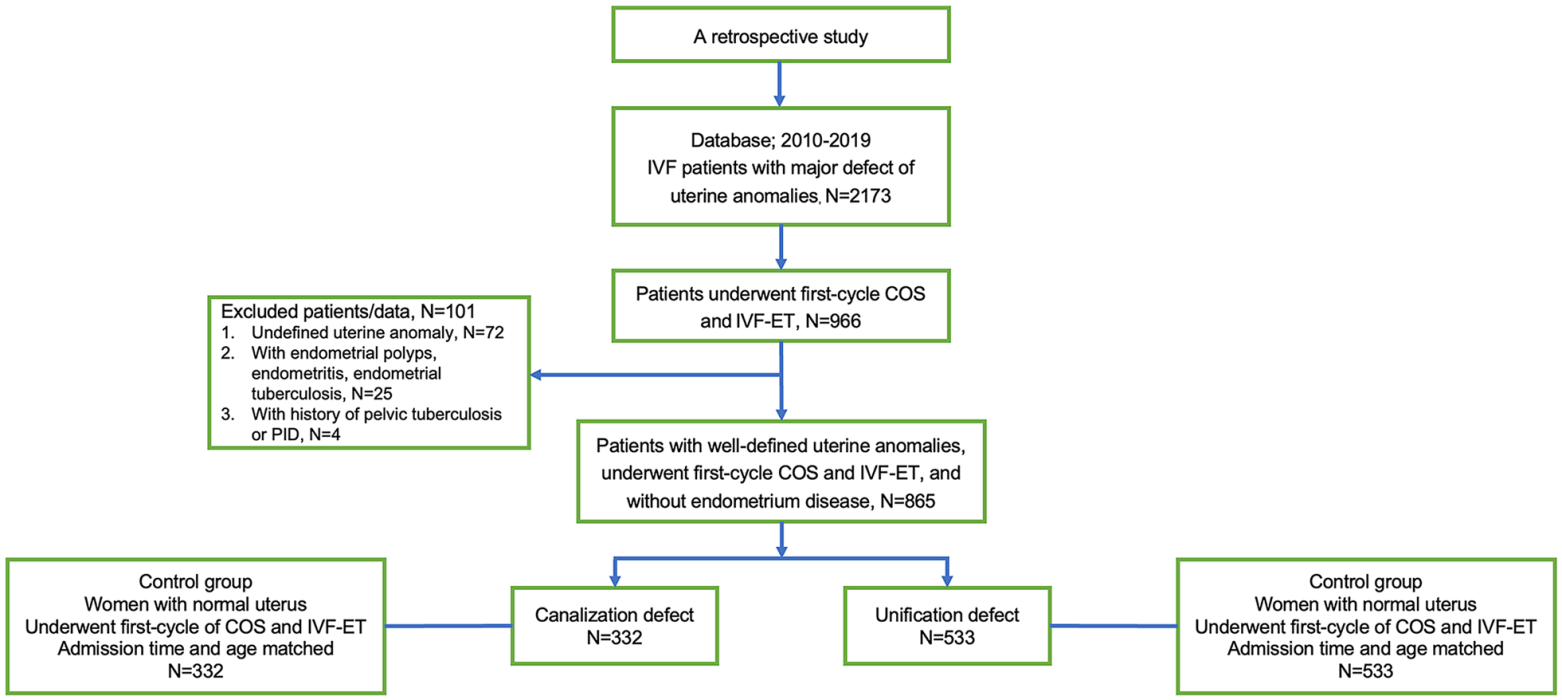
Flow chart of the study

Among the 865 women with uterine anomalies, 332 (38.4%) were diagnosed with septate uterus, 23 (2.7%) were delineated as bicornuate uterus, 79 (9.1%) were diagnosed with didelphus uterus, and 431 (49.8%) were diagnosed with unicornuate uterus. According to abnormal fusion or septal absorption of the Müllerian ducts, we classified the cohort of uterine anomalies into two groups: the canalization defect group (including septate and subseptate uterus, N = 332) and the unification defect group (including bicornuate, didelphus, and unicornuate uterus, N = 533). According to the admission time and age, we randomly retrieved clinical data of women with normal uteri and underwent first cycle IVF-ET from the database in 1:1 match (Fig. 1).

### Baseline characteristics

The baseline characteristics of these study groups are summarized in Table 1. Compared with control group, women with canalization defects had similar age, infertility type, infertility duration, previous pregnancy, previous delivery, body mass index (BMI), antral follicle count (AFC), and follicle stimulating hormone (FSH) level and cause of infertility. Compared with women with a normal uterus, women with a unification defect had higher serum FSH level (6.97 ± 6.91 vs 6.22 ± 2.99 IU/ml, P = 0.017) and higher proportion of tubal factor caused infertility (31.9% vs 20.8%) and lower proportion of male factor caused infertility (17.4% vs 25.1%) (P < 0.001). The cohort characterized by canalization defect exhibited a spontaneous miscarriage rate of 6.3% (21/332) for history of 2 or more spontaneous miscarriages, while the cohort afflicted with canalization defect displayed a rate of 3.0% (16/533) for similar occurrences, both of which were markedly greater when compared to the control group (P < 0.05). It is important to note that patients in the control group with a history of multiple miscarriages were all classified as having unexplained etiologies, whereas within the uterine abnormality group, no factors other than uterine abnormalities were identified as potential causes of recurrent miscarriages. While age, infertility type, infertility duration, previous pregnancy, previous delivery, BMI and AFC were similarly distributed between these two groups. The characteristics of study participants are complete, without any missing data.

**Table 1:** Patient characteristics at baseline

	Controls	Canalization defect		Controls	Unification defect	
			P value			P value
No. of patients	332	332		533	533	
Age (years old)	32.0 ± 4.3	32.1 ± 4.5	0.947	31.1 ± 4.0	31.0 ± 4.3	0.620
Infertility type			0.162			0.576
Primary infertility	183 (55.1%)	165 (49.7%)		310 (58.2%)	319 (59.8%)	
Secondary infertility	149 (44.9%)	167 (50.3%)		223 (41.8%)	214 (40.2%)	
Infertility duration	3 (2, 5)	3 (2, 6)	0.453	3 (2,5)	3 (2, 5)	0.218
Previous pregnancy			0.201			0.901
0	198 (59.6%)	182 (54.8%)		341 (64.0%)	333 (62.5%)	
1	77 (23.2%)	79 (23.8%)		118 (22.1%)	121 (22.7%)	
2	30 (9.0%)	47 (14.2%)		42 (7.9%)	50 (9.4%)	
≥ 3	27 (8.1%)	24 (7.2%)		32 (6.0%)	29 (5.4%)	
Previous delivery			0.255			0.220
0	313 (94.3%)	318 (95.8%)		509 (95.5%)	507 (95.1%)	
1	18 (5.4%)	11 (3.3%)		24 (4.5%)	23 (4.3%)	
2	1 (0.3%)	3 (0.9%)		0 (0.0%)	3 (0.6%)	
Spontaneous miscarriage			0.002			0.048
0	315 (94.9%)	296 (89.2%)		508 (95.3%)	500 (93.8%)	
1	13 (3.9%)	15 (4.5%)		20 (3.8%)	17 (3.2%)	
≥ 2	4 (1.2%)	21 (6.3%)		5 (0.9%)	16 (3.0%)	
BMI (kg/m^2^)	22.68 ± 3.63	23.06 ± 3.49	0.141	22.4 ± 3.96	22.93 ± 3.77	0.806
AFC	11 (7, 15)	10 (7, 13.75)	0.122	10 (6, 14)	10 (6, 14)	0.892
Baseline serum FSH (IU/ml)	6.33 ± 2.78	6.42 ± 2.69	0.668	6.22 ± 2.99	6.97 ± 6.91	0.017
Cause of infertility			0.276			0.000
Unexplained reason	77 (22.0%)	82 (24.7%)		111 (20.8%)	120 (22.5%)	
Both factor	66 (19.9%)	71 (21.4%)		106 (19.9%)	101 (18.9%)	
Male factor	85 (25.6%)	67 (20.2%)		134 (25.1%)	93 (17.4%)	
Female factor						
Tubal factor	66 (19.9%)	80 (24.1%)		111 (20.8%)	170 (31.9%)	
Decreased ovarian reserve	14 (4.2%)	8 (2.4%)		20 (3.8%)	15 (2.8%)	
Ovulation dysfunction	18 (5.4%)	19 (5.9%)		38 (7.1%)	26 (4.9%)	
Endometriosis	10 (3.0%)	5 (1.5%)		13 (2.4%)	8 (1.5%)	
Complicated with myoma	12 (3.6%)	20 (6.0%)	0.147	19 (3.6%)	13 (2.4%)	0.282

### Outcome of controlled ovarian stimulation

The characteristics of the assisted reproduction cycle of these groups are summarized in Table 2. Compared with women with normal uteri, women with canalization defects were less frequently used ICSI (32.5% vs. 41.9%, P = 0.002) and transferred less double cleavage embryos (74.7% vs 83.7%), more triple cleavage embryos (8.7% vs 3.3%) and more double blastocysts (5.4% vs 1.2%) (P = 0.012). The stimulation type, total Gn dose, total number of oocytes retrieved and high-quality embryo transferred were comparable between these two groups.

**Table 2:** Outcomes of controlled ovarian hyperstimulation

	Controls	Canalization defect		Control	Unification defect	
No. of patients	332	332		533	533	
Stimulation type			0.304			0.101
Downregulation	172 (51.8%)	151 (45.5%)		253 (47.5%)	226 (42.4%)	
Antagonist	137 (41.3%)	152 (45.8%)		253 (47.5%)	268 (50.3%)	
Agonist	23 (6.9%)	28 (8.4%)		27 (5.1%)	36 (6.8%)	
Microstimulation	0 (0.0%)	1 (0.3%)		0 (0.0%)	3 (0.6%)	
Total Gn dose (IU)	2443.7 (1800,3431)	2600 (1875,3450)	0.324	2475 (1800, 3318)	2475 (1800,3312.5)	0.349
Total number of oocytes retrieved	11 (7,14)	11 (7,15)	0.120	10 (6.5, 14)	10 (7,14)	0.134
IVF technique used			0.002			0.000
Classic IVF	190 (57.2%)	209 (63.0%)		323 (60.0%)	401 (75.2%)	
ICSI	139 (41.9%)	108 (32.5%)		207 (38.8%)	119 (22.3%)	
HALF	3 (0.9%)	15 (4.5%)		3 (0.6%)	13 (2.4%)	
Embryos/blastocysts transferred			0.012			0.000
Single cleavage embryo	29 (8.7%)	33 (9.9%)		37 (6.9%)	124 (23.3%)	
Double cleavage embryos	278 (83.7%)	248 (74.7%)		458 (85.9%)	259 (48.6%)	
Triple cleavage embryos	11 (3.3%)	29 (8.7%)		11 (2.1%)	127 (23.8%)	
Single blastocyst	10 (3.0%)	4 (1.2%)		19 (3.6%)	7 (1.3%)	
Double blastocysts	4 (1.2%)	18 (5.4%)		8 (1.5%)	16 (3.0%)	
High quality embryos (%)^a^	1415 (93.4%)	312 (95.2%)	0.316	493 (93.4%)	477 (92.1%)	0.423

^a^High-quality Day 3 embryos were defined as embryos that were developed from 2PN, reached the 5- to 8-cell stage, and had cytoplasmic fragmentation occupying < 30% of the embryo surface and equal-sized blastomeres

Compared with women with a normal uterus, women with unification defects were also less frequently used ICSI (22.3% vs. 38.8%, P < 0.001) and transferred less double cleavage embryos (48.6% vs 85.9%), more triple cleavage embryos (23.8% vs 2.1%) and more double blastocysts (3.0% vs 1.5%) (P < 0.001). The stimulation type, total Gn dose, total number of oocytes retrieved and high-quality embryos were comparable between these two groups.

### Live birth and secondary outcomes

When compared with women with a normal uterus, women with canalization defects were less likely to experience live birth [84/332 (25.3%) vs 128/332 (38.6%), RR: 0.647, 95% CI 0.513–0.815, P < 0.001], as were women with unification defects [132/533 (24.8%) vs 219/533 (41.1%), RR: 0.713, 95% CI 0.586–0.868, P = 0.001] (Table 3). Canalization defects also resulted in a lower frequency of singleton live births than normal uterus (19.6% vs.25.9%), for a rate ratio of 0.706 (95% CI, 0.532 to 0.938; P = 0.016), as well as a reduced rate of twin live births [19/332 (5.7%) vs 42/332 (12.7%), RR: 0.378, 95% CI 0.221–0.646, P < 0.001]. Similarly, the fusion disorder group also exhibited a lower frequency of singleton live births than normal uterus (22.0% vs. 27.2%), for a rate ratio of 0.712 (95% CI, 0.575 to 0.883; P = 0.002) and a lower frequency of twin live births (2.8% vs. 13.9%, RR: 0.194, 95% CI 0.113–0.333, P < 0.001). Similarly, either women with canalization defects or unification defects were less likely to experience clinical pregnancy than women with normal uteri [126/332 (38.0%) vs 206/332 (62.0%), RR: 0.829, 95% CI 0.690–0.997, P = 0.046; 182/533 (34.1%) vs 263/533 (49.1%), RR: 0.813, 95% CI 0.695–0.952, P = 0.010 respectively]. Women with canalization defects were also more likely to experience spontaneous first-trimester pregnancy loss than women with normal uteri [25/126 (19.8%) vs 11/206 (5.3%), RR: 2.716, 95% CI 1.393–5.295, P = 0.003], as were women with unification defects [36/182 (19.8%) vs 20/263 (7.6%), RR: 3.288, 95% CI 1.776–6.085, P < 0.001]. Compared with women with normal uteri, both women with canalization defects and women with unification defects all experienced a similar likelihood of ectopic pregnancy, second-trimester pregnancy loss and preterm birth. Whether bearing singleton or multiple pregnancies, uterine anomalies demonstrated no statistically significant disparities in rates of second-trimester pregnancy loss and preterm birth when compared to the control group (Table 3).

**Table 3:** Reproductive outcomes of first-cycle IVF-ET

	Canalization defect vs. Control				Unification defect vs. control			
	Control	Canalization defect	RR (95%CI)	^a^*P* value	Control	Unification defect	RR (95%CI)	^a^*P* value
Clinical pregnancy	206 (62.0%)	126 (38.0%)	0.829 (0.690,0.997)	0.046	263 (49.1%)	182 (34.1%)	0.813 (0.695,0.952)	0.010
Ectopic pregnancy	3 (1.4%)	5 (4.0%)	1.992 (0.486,8.168)	0.543	6 (2.3%)	5 (2.7%)	1.247 (0.309,5.037)	1.247
First-trimester pregnancy loss	11 (5.3%)	25 (19.8%)	2.716 (1.393,5.295)	0.003	20 (7.6%)	36 (19.8%)	3.288 (1.776,6.085)	0.000
Second-trimester pregnancy loss	4 (1.9%)	8 (6.3%)	2.091 (0.627,6.978)	0.230	11 (4.2%)	6 (3.3%)	0.854 (0.288,2.532)	0.776
Singleton	3 (1.4%)	4 (3.2%)	1.618 (0.370,7.086)	0.523	6 (2.3%)	5 (2.7%)	1.176 (0.365, 3.793)	0.786
Twin	1 (0.5%)	4 (3.2%)	3.621 (0.382,34.34)	0.262	5 (1.9%)	1 (0.5%)	0.288 (0.034, 2.411)	0.253
Live birth	128 (38.6%)	84 (25.3%)	0.647 (0.513,0.815)	0.000	219 (41.1%)	132 (24.8%)	0.713 (0.586,0.868)	0.001
Singleton	86 (25.9%)	65 (19.6%)	0.706 (0.532, 0.938)	0.016	145 (27.2%)	117 (22.0%)	0.712 (0.575, 0.883)	0.002
Twin	42 (12.7%)	19 (5.7%)	0.378 (0.221,0.646)	0.000	74 (13.9%)	15 (2.8%)	0.194 (0.113,0.333)	0.000
Preterm birth	23 (17.9%)	15 (17.8%)	0.875 (0.471,1.629)	0.674	42 (19.2%)	26 (19.7%)	1.106 (0.619,1.975)	0.733
Singleton	10 (7.8%)	8 (9.5%)	1.147 (0.411, 3.202)	0.794	15 (6.9%)	17 (12.9%)	1.462 (0.612, 3.442)	0.385
Twin	13 (10.1%)	7 (8.3%)	0.993 (0.287, 3.436)	0.991	27 (12.3%)	9 (6.8%)	0.310 (0.074, 1.299)	0.109

^a^Adjusted for age, BMI, infertility duration, stimulation type, IVF technique, total number of oocytes retrieved and embryos/blastocysts transferred

Next, we did subgroup analysis by type of unification defect. We also did a 1:1 matched selection of control group for each subtype of unification defect. The comparison of baseline characteristics between unicornuate uterus, bicornuate uterus or uterus didelphys and control group were presented in Additional file 2: Table S1. Women with unicornuate uterus had lower clinical pregnancy rate [139/431 (32.3%) vs 212/431 (49.2%), P < 0.001], lower live-birth rate [101/431 (23.4%) vs 181/431 (42.0%), P < 0.001], and higher first-trimester pregnancy loss [26/139 (18.7%) vs 12/212 (5.7%), P < 0.001] than did controls. While they didn’t experience higher rates of preterm birth [19/101 (18.8%) vs 31/181 (17.8%), P = 0.722]. Women with uterus didelphys also experienced lower clinical pregnancy rate [30/74 (40.5%) vs 43/74 (58.1%), P = 0.033], lower live-birth rate [21/74 (28.4%) vs 39/74 (52.7%), P = 0.003], and higher first-trimester pregnancy loss [8/30 (26.7%) vs 2/43 (4.7%), P = 0.013]. Women with bicornuate uterus had similar reproductive outcomes with those with a normal uterus (Table 4).

**Table 4:** Reproductive outcomes of first-cycle IVF-ET by subtype of unification defecations

	Unicornuate uterus (N = 431)			Bicornuate uterus (N = 23)			Uterus didelphys (N = 74)		
		Controls	P value		Controls	P value		Controls	P value
Clinical pregnancy	139 (32.3%)	212 (49.2%)	< 0.001	12 (52.2%)	13 (56.5%)	0.767	30 (40.5%)	43 (58.1%)	0.033
Ectopic pregnancy	4 (2.9%)	5 (2.4%)	0.765	0 (0.0%)	0 (0.0%)	–	1 (3.3%)	1 (2.3%)	1.000
First-trimester pregnancy loss	26 (18.7%)	12 (5.7%)	< 0.001	2 (16.7%)	2 (15.4%)	1.000	8 (26.7%)	2 (4.7%)	0.013
Second-trimester pregnancy loss	6 (4.3%)	9 (4.2%)	0.974	0 (0.0%)	1 (7.7%)	1.000	0 (0.0%)	1 (2.3%)	1.000
Live birth	101 (23.4%)	180 (41.8%)	< 0.001	9 (40.9%)	10 (43.5%)	0.862	21 (28.4%)	39 (52.7%)	0.003
Preterm birth	19 (18.8%)	31 (17.8%)	0.722	1 (11.1%)	0 (0%)	0.474	5 (23.8%)	4 (10.3%)	0.306

## Discussion

The major findings of this investigation demonstrate that women with canalization defect and women with unification defect all were less likely to achieve clinical pregnancy and live birth and were more likely to achieve spontaneous early pregnancy loss after IVF-ET when compared with women with normal uterus. 

### Strengths and limitations

Our present work has some strengths of note. First, we classified the major defect of uterine malformations into two categories, unification defects and canalization defects, highlighting an at-risk population that needs to be an adequate clinical explanation of poor reproductive performance or possible interventions. Second, our study had a large sample size, and we adjusted for more possible confounders, which guaranteed statistical power. Third, we selected the control group according to the age and admission time matched, which enable the baseline characteristics between uterine anomalies and control group more comparable. In addition, the observational study design enabled us to thoroughly describe baseline characteristics as well as assisted reproductive outcomes.

Our study also has a series of limitations. First, some relevant characteristics, such as cause of infertility, IVF technique used, and embryo transfer, were significantly different between the control group and uterine anomaly group. The uterine anomaly group underwent more classic IVF (70.5% vs 59.3%) and less ICSI (26.2% vs 40%). These discrepancies maybe due to the uterine anomaly itself. Since these factors were not fully matched, we adjusted for some factors, including age, BMI, infertility duration, stimulation type, total number of oocytes retrieved and embryos/blastocysts transferred, in the log binomial model. The conclusions were consistent with the univariate analysis. Another limitation is that, a total of 209 women were transferred three embryos/blastocysts, which is not currently recommeneded. In routine clinical practice in our hospital, the number of embryos transferred was influenced by the stage of embryos transferred, the total number of embryos available in a cycle and uterine factors, such as uterine anomalies, history of cesarean, history of cervical conization and so on. The proportions of transferring triple cleavage embryos in the control group, canalization defect group and unification defect group were 2.5%, 8.7% and 23.8%, respectively. This may be attributed to the retrospective nature of the study, spanning the past decade during which there was no consensus on the number of embryos to be transplanted. Some reproductive specialists aimed to enhance the success rates of IVF in patients with uterine anomalies, thus exhibiting a propensity for transferring a greater number of embryos. The slightly high proportion of transferring triple cleavage embryos may limit the generality of our conclusions. Other limitations include the possible heterogeneity in the diagnostic approach across the last decades. In addition, obstetrical outcomes were not fully documented because those women were followed up by telephone.

### Interpretations

Congenital uterine anomalies are uterine malformations caused by abnormal development of embryonic bilateral Müllerian duct, which can be variable from complete absence of a uterus through to more subtle anomalies.

Congenital uterine anomalies were found to have detrimental effects on obstetric outcomes, including preterm birth, cervical insufficiency, prelabor rupture of membranes, fetal malpresentation, fetal growth restriction, placental abruption, placenta previa, placental retention, and cesarean birth [23]. The assisted reproductive outcomes of women with congenital uterine anomalies have been discussed by several retrospective and prospective studies. Consistent with previous studies, we also found that major defects (including septate uterus, unicornuate uterus, bicornuate uterus, didelphic uterus) decrease clinical pregnancy rate and live birth rate of IVF/ICSI-ET.

Some studies found that uterine malformation had little effect on the outcome of assisted reproduction, and the reason was that the arcuate uterus occupied a large proportion [6]. With the exception of the arcuate uterus, significantly lower clinical pregnancy rate and live birth birth rate were found in women with major defects [6]. Currently, evidence suggests that the arcuate uterus is an incidental finding without any appreciable impact on fertility and a previous study showed that arcuate uterus had no impact on IVF outcomes after euploid embryo transfer [24]. In addition, the European Society of Human Reproduction and Embryology (ESHRE) and the European Society for Gynecological Endoscopy (ESGE) consensus on the classification of female genital tract congenital anomalies established in 2013 had cancelled the definition “arcuate uterus” [25]. Therefore, it is inappropriate to study uterine malformation as a whole when studying the influence of uterine malformation on reproduction. However, if the effect of each uterine malformation on IVF outcome was studied separately, bias might appear because of the low incidence of uterine malformation and the small sample size of individual uterine malformations. Based on this, our study divided the uterine anomaly into canalization defect and unification defect according to the developmental process of embryonic Müllerian ducts [13], which would be more conducive to clinical consultation.

Similar to reproductive outcomes of previous study in first cycle FET, we also found canalization defect, including septal uterus and subseptal uterus, appeared to experience poor reproductive performance, with a reduced live birth rate, clinical pregnancy rate and increased pregnancy loss [10]. While no increased preterm birth rate was found, which was different from the conclusion found in nature pregnancy [3], probably because in our cohort most of the patients with septate uterus underwent surgical treatment. Based on the poor reproductive performance, several studies have compared the reproductive outcomes between septum resection and expectant management for women with a septate uterus and drawn contrary conclusions. Retrospective studies focused on the IVF/ICSI outcomes in women before and after hysteroscopic resection of a uterine septum compared to normal controls found that lower pregnancy rate and higher pregnancy loss rate in the before hysteroscopic metroplasty group than that in control group and similar pregnancy rate and pregnancy loss rate in after hysteroscopic metroplasty group than that in control group [26, 27]. However, the randomized uterine septum transsection trial (TRUST) found that hysteroscopic septum resection did not improve reproductive outcomes in women with a septate uterus [28]. Limited by the small sample size and inadequate follow-up period, more studies are need to resolve the issue of whether or not to resect uterine septum [28].

Similar to canalization defects, unification defects were also found to decrease the clinical pregnancy rate, live birth rate and increase first-trimester pregnancy loss rate after IVF-ET. Previous studies found that unification defects increased the risk of preterm birth [29]. The laterality of placental location in pregnant women with Müllerian anomaly leads to discordant uterine artery velocity flow waveforms, which could be implicated in the pathophysiologic mechanism of preterm birth [30]. But in our study, we found the incidence of preterm delivery in women with unification defects was comparable to that in control group. The discrepancy may be due to that more single cleavage embryo was transferred in unification group. Subgroup analysis addressed similar conclusion in women with unicornuate uterus and uterus didelphys. While bicornuate uterus was found to experienced similar reproductive outcomes with controls. There could be no difference at all or the difference was so small that we didn't have a large enough sample size to detect it. In the future, a prospective randomized controlled study comparing surgical and non-surgical interventions for specific subgroups of uterine abnormalities in the context of IVF with euploid embryo transfer to make a conclusive information useful for evidence dictation medical management of those patients.

## Conclusion

In summary, this large study demonstrated that women with canalization defect and women with unification defect were less likely to achieve clinical pregnancy and live birth and were more likely to experience spontaneous early pregnancy loss after IVF-ET when compared with women with normal uterus.

### Supplementary Information

**Additional file 1. **Controlled ovarian hyperstimulation protocols.

**Additional file 2. Table S1.** Characteristics of unicornuate, bicornuate and didelphys uterus and their matched control groups.

## Data Availability

Researchers who want access to datasets for replication should apply to kangjiasdu@163.com.

## Abbreviations

AFC: Antral follicle count
AFS: American Fertility Society
ART: Assisted reproduction technology
BMI: Body Mass Index
COS: Controlled ovarian stimulation
ESHRE: European Society of Human Reproduction and Embryology
ESGE: European Society for Gynecological Endoscopy
ET: Embryo transfer
FET: Frozen thawed embryo transfer
FSH: Follicle stimulating hormone
ICSI: Intracytoplasmic sperm injection
IVF: In vitro fertilization
TRUST: The randomized uterine septum transection trial
2D: Two-dimensional
3D: Three-dimensional
