# Supplementary material for: Impact of congenital uterine anomalies on reproductive outcomes of IVF/ICSI-embryo transfer: a retrospective study
Source: Eur J Med Res. 2024 Jan 11;29:48. doi: 10.1186/s40001-023-01544-2 (PMC10782742; doi:10.1186/s40001-023-01544-2)
Supplement: Supplementary file 1 — Additional file 1. Controlled ovarian hyperstimulation protocols. [file 40001_2023_1544_MOESM1_ESM.docx]

**Additional file 1 Controlled ovarian hyperstimulation protocols**

All women who underwent IVF/ICSI cycles received controlled ovarian hyperstimulation protocols as previously described[1, 2]. Briefly, the downregulation protocol included the gonadotrophin-releasing hormone (GnRH) agonist long protocol and the ultralong protocol. In the long protocol, patients received pituitary downregulation by midluteal administration of a GnRH agonist, 0.1 mg triptorelin acetate daily injection, or 1.3 mg/1.8 mg triptorelin once injection following a hormone test 14 days later. In the GnRH agonist ultralong protocol, patients undertook ovarian stimulation that was induced by the rFSH protocol initial between Day 28 and Day 30 of their menstrual cycle following pituitary downregulation by 3.75 mg of triptorelin acetate or leuprorelin acetate on the first day of that cycle. In the antagonist protocol, GnRH antagonist (ganirelix acetate or cetrorelix acetate) was given beginning on Day 5 of rFSH injection, or when the leading follicle reached 10 mm, to the day of human chorionic gonadotrophin (hCG) administration. In the agonist protocol, patients were administered GnRH agonist from the second day of their menstrual cycle onward. COH was given to those patients by recombinant FSH (rFSH) or human menopausal gonadotropin (HMG) in different flexible protocols. In the microstimulation protocol, 2.5 mg letrozole or 50 mg clomiphene citrate was administered daily from Day 2 to 6 of menstruation, and 150 IU of recombinant FSH was initiated from Day 5.

In all treatment protocols, once at least two leading follicles reached a size of ≥18 mm, 5000 to 10 000 IU hCG or 250 μg recombinant hCG (r-hCG) was administered to trigger ovulation, and ovum collection was performed between 36 and 38 hours later. Oocytes were fertilized by either conventional IVF or intracytoplasmic sperm injection. Embryo transfer was accomplished on day 3 or 5 of the cycle according to the Code of Practice for Assisted Reproductive Technology developed by the Ministry of Health of the People's Republic of China. High-quality embryos were defined as embryos that were developed from 2PN, had more than 5 blastomeres, had a size difference less than 20%, and had fragmentation less than 30%[1, 3]. The luteal phase was supported from the day of ET to maintain lutein function through the 10th week of pregnancy.

1. Wang H, Gao H, Chi H, Zeng L, Xiao W, Wang Y, et al. Effect of Levothyroxine on Miscarriage Among Women With Normal Thyroid Function and Thyroid Autoimmunity Undergoing In Vitro Fertilization and Embryo Transfer: A Randomized Clinical Trial. JAMA. 2017;318:2190-8.

2. Yang R, Niu ZR, Chen LX, Liu P, Li R, Qiao J. Analysis of related factors affecting cumulative live birth rates of the first ovarian hyperstimulation in vitro fertilization or intracytoplasmic sperm injection cycle: a population-based study from 17,978 women in China. Chin Med J (Engl). 2021;134:1405-15.

3. Song XL, Lu CL, Zheng XY, Nisenblat V, Zhen XM, Yang R, et al. Enhancing the scope of in vitro maturation for fertility preservation: transvaginal retrieval of immature oocytes during endoscopic gynaecological procedures. Hum Reprod. 2020;35:837-46.
