# Supplementary material for: Impact of congenital uterine anomalies on reproductive outcomes of IVF/ICSI-embryo transfer: a retrospective study
Source: Eur J Med Res. 2024 Jan 11;29:48. doi: 10.1186/s40001-023-01544-2 (PMC10782742; doi:10.1186/s40001-023-01544-2)
Supplement: Supplementary file 2 — Additional file 2. Table S1. Characteristics of unicornuate, bicornuate and didelphys uterus and their matched control groups. [file 40001_2023_1544_MOESM2_ESM.docx]

Additional file 2: Characteristics of unicornuate, bicornuate and didelphys uterus and their matched control groups

Table S1 Characteristics of unicornuate, bicornuate and didelphys uterus and their matched control groups

|  | **Unicornuate uterus** | | | **Bicornuate uterus** | | | **Uterus didelphys** | | |
| --- | --- | --- | --- | --- | --- | --- | --- | --- | --- |
|  |  | **Control** | **P value** |  | **Control** | **P value** |  | **Control** | **P value** |
| **No. of patients** | 431 | 431 |  | 23 | 23 |  | 74 | 74 |  |
| **Age (years old)** | 30.9±4.1 | 31.3±3.8 | 0.102 | 31.0±4.0 | 31.0±4.0 | 1.000 | 31.0±5.3 | 31.0±4.9 | 0.974 |
| **Infertility type** |  |  | 0.301 |  |  | 0.127 |  |  | 0.868 |
| Primary infertility | 257(59.6%) | 242(56.1%) |  | 17(73.9%) | 12(52.2%) |  | 41(55.4%) | 42(56.8%) |  |
| Secondary infertility | 174(40.4%) | 189(43.9%) |  | 6(26.1%) | 11(47.8%) |  | 33(44.6%) | 32(43.2%) |  |
| **Infertility duration** | 3(2, 5) | 3(2, 5) | 0.018 | 3(1, 7) | 3(2, 5) | 0.257 | 3(2,5) | 3(2, 5) | 0.710 |
| **Previous pregnancy** |  |  | 0.609 |  |  | 0.503 |  |  | 0.534 |
| 0 | 270(62.6%) | 269(62.4%) |  | 17(73.9) | 13(56.5%) |  | 42(56.8%) | 44(59.5%) |  |
| 1 | 100(23.2%) | 95(22.0%) |  | 4(17.4%) | 7(30.4%) |  | 17(23.0%) | 16(21.6%) |  |
| 2 | 40(9.3%) | 35(8.1%) |  | 2(8.7%) | 2(8.7%) |  | 8(10.8%) | 8(10.8%) |  |
| ≥3 | 21(4.9%) | 32(7.4%) |  | 0(0.0%) | 1(4.3%) |  | 7(9.5%) | 6(8.1%) |  |
| **Previous delivery** |  |  | 0.139 |  |  | - |  |  | 0.061 |
| 0 | 412(95.6%) | 405(94.0%) |  | 23(100%) | 23(100%) |  | 67(90.5%) | 73(98.6%) |  |
| 1 | 17(3.9%) | 26(6.0%) |  | 0 | 0 |  | 6(8.1%) | 1(1.4%) |  |
| 2 | 2(0.5%) | 0(0.0%) |  | 0 | 0 |  | 1(1.4%) | 0(0.0%) |  |
| **BMI (kg/m^2^)** | 22.9±3.8 | 22.9±4.1 | 0.744 | 22.1±3.5 | 22.6±3.1 | 0.244 | 21.8±3.8 | 22.7±3.3 | 0.379 |
| **AFC** | 9(6, 14) | 10(6,14) | 0.250 | 11 (7,16) | 11(6, 15) | 1.000 | 10(7, 14) | 10(6, 14) | 0.919 |
| **Baseline FSH (IU/ml)** | 6.96±7.55 | 6.12±2.91 | 0.051 | 6.6±3.3 | 5.7±3.3 | 0.551 | 6.25±2.57 | 6.63±2.53 | 0.141 |
| **Cause of infertility** |  |  | 0.000 |  |  | 0.872 |  |  | 0.858 |
| Unexplained reason | 98(22.7%) | 82(19.0%) |  | 4(17.4%) | 7(30.4%) |  | 18(24.3%) | 18(24.3%) |  |
| Both factor | 78(18.1%) | 90(20.9%) |  | 4(17.4%) | 5(21.7%) |  | 18(24.3%) | 12(16.2%) |  |
| Male factor | 70(16.2%) | 112(26.0%) |  | 6(26.1%) | 5(21.7%) |  | 15(20.3%) | 19(25.7%) |  |
| Female factor |  |  |  |  |  |  |  |  |  |
| Tubal factor | 146(33.9%) | 94(21.8%) |  | 5(21.7%) | 4(17.4%) |  | 17(23.0%) | 18(24.3%) |  |
| DOR | 13(3.0%) | 17(3.9%) |  | 1(4.3%) | 1(4.3%) |  | 1(1.4%) | 1(1.4%) |  |
| PCOS | 22(5.1%) | 24(5.6%) |  | 2(8.7%) | 1(4.3%) |  | 2(2.7%) | 4(5.4%) |  |
| Endometriosis | 4(0.9%) | 12(2.8%) |  | 1(4.3%) | 0(0.0%) |  | 3(4.1%) | 2(2.7%) |  |
| **Complicated with myoma** | 15(3.5%) | 9(2.1%) | 0.214 | 1(4.3%) | 1(4.3%) | 1.000 | 3(4.1%) | 5(6.8%) | 0.716 |
| **Stimulation type** |  |  | 0.048 |  |  | 0.649 |  |  | 0.223 |
| Downregulation | 175(40.6%) | 200(46.4%) |  | 13(56.5%) | 10(43.5%) |  | 35(47.3%) | 43(58.1%) |  |
| Antagonist | 225(52.2%) | 214(49.7%) |  | 8(34.8%) | 11(47.8%) |  | 34(45.9%) | 30(40.5%) |  |
| Agonist | 29(6.7%) | 17(3.9%) |  | 2(8.7%) | 2(8.7%) |  | 4(5.4%) | 1(1.4%) |  |
| Microstimulation | 2(0.5%) | 0(0.0%) |  | 0(0.0%) | 0(0.0%) |  | 1(1.4%) | 0(0.0%) |  |
| **Total Gn dose (IU)** | 2475±1223 | 2611±1210 | 0.429 | 2887±805 | 2850±1251 | 0.708 | 2475±1188 | 2537±1187 | 0.764 |
| **Total number of oocytes retrieved** | 10(6, 14) | 10(7, 14) | 0.221 | 10(4, 17) | 13(8,16) | 0.461 | 11(7, 15) | 12(7, 15) | 0.945 |
| **IVF technique used** |  |  | 0.000 |  |  | 0.022 |  |  | 0.438 |
| Classic IVF | 332(77.0%) | 262(60.8%) |  | 18(78.3%) | 11(47.8%) |  | 49(66.2%) | 46(62.2%) |  |
| ICSI | 90(20.9%) | 162(37.6%) |  | 5(21.7%) | 10(43.5%) |  | 22(29.7%) | 27(36.5%) |  |
| HALF | 9(2.1%) | 7(1.6%) |  | 0(0.0%) | 2(8.7%) |  | 3(4.1%) | 1(1.4%) |  |
| **Embryos/blastocysts transferred** |  |  | 0.000 |  |  | 0.020 |  |  | 0.104 |
| Single cleavage embryo | 104(24.1%) | 36(8.4%) |  | 7(30.4%) | 1(4.3%) |  | 11(14.9%) | 7(9.5%) |  |
| Double cleavage embryos | 197(45.7%) | 363(84.2%) |  | 12(52.2%) | 21(91.3%) |  | 48(64.9%) | 56(75.7%) |  |
| Triple cleavage embryos | 12(2.8%) | 9(2.1%) |  | 1(4.3%) | 0(0.0%) |  | 3(4.1%) | 7(9.5%) |  |
| Single blastocyst | 116(26.9%) | 19(4.4%) |  | 2(8.7%) | 0(0.0%) |  | 8(10.8%) | 2(2.7%) |  |
| Double blastocysts | 2(0.5%) | 4(0.9%) |  | 1(4.3%) | 1(4.3%) |  | 4(5.4%) | 2(2.7%) |  |
| **High quality embryos (%)** | 386(92.1%) | 404(94.2%) | 0.237 | 21(91.3%) | 22(95.7%) | 1.000 | 66(93.0%) | 71(97.3%) | 0.416 |
